# Extracytoplasmic function (ECF) sigma factor σ^F^ is involved in *Caulobacter crescentus* response to heavy metal stress

**DOI:** 10.1186/1471-2180-12-210

**Published:** 2012-09-18

**Authors:** Christian Kohler, Rogério F Lourenço, Gabriela M Avelar, Suely L Gomes

**Affiliations:** 1 Departamento de Bioquímica, Instituto de Química, Universidade de São Paulo, Av. Prof. Lineu Prestes, 748, 05508-000, São Paulo, SP, Brazil; 2 Present address: Friedrich Loeffler Institut for Medical Microbiology, Greifswald, Germany

**Keywords:** Stress response, ECF sigma factor σ^F^, Chromium, Cadmium, Caulobacter crescentus

## Abstract

**Background:**

The α-proteobacterium *Caulobacter crescentus* inhabits low-nutrient environments and can tolerate certain levels of heavy metals in these sites. It has been reported that *C. crescentus* responds to exposure to various heavy metals by altering the expression of a large number of genes.

**Results:**

In this work, we show that the ECF sigma factor σ^F^ is one of the regulatory proteins involved in the control of the transcriptional response to chromium and cadmium. Microarray experiments indicate that σ^F^ controls eight genes during chromium stress, most of which were previously described as induced by heavy metals. Surprisingly, σ^F^ itself is not strongly auto-regulated under metal stress conditions. Interestingly, σ^F^-dependent genes are not induced in the presence of agents that generate reactive oxygen species. Promoter analyses revealed that a conserved σ^F^-dependent sequence is located upstream of all genes of the σ^F^ regulon. In addition, we show that the second gene in the *sigF* operon acts as a negative regulator of σ^F^ function, and the encoded protein has been named NrsF (Negative regulator of sigma F). Substitution of two conserved cysteine residues (C131 and C181) in NrsF affects its ability to maintain the expression of σ^F^-dependent genes at basal levels. Furthermore, we show that σ^F^ is released into the cytoplasm during chromium stress and in cells carrying point mutations in both conserved cysteines of the protein NrsF.

**Conclusion:**

A possible mechanism for induction of the σ^F^-dependent genes by chromium and cadmium is the inactivation of the putative anti-sigma factor NrsF, leading to the release of σ^F^ to bind RNA polymerase core and drive transcription of its regulon.

## Background

Several heavy metals play important roles as trace elements in the metabolism of all kingdoms of life. Whether a trace element is useful or harmful depends on its concentration. Particularly, chromium and cadmium are known to be much more toxic than useful for most microorganisms [1,2]. Chromium is commonly present in solutions as chromate and dichromate oxyanions (Cr(VI)), the most redox-reactive and soluble forms of the metal [3]. Due to its similar chemical structure to sulfate anions, chromate crosses membranes via sulfate uptake systems [4]. On the other hand, cadmium is a non-redox-reactive metal with high affinity for thiol groups [1,2]. Once inside cells, chromate, dichromate and cadmium exert their toxic effects by directly damaging cellular components and by inducing oxidative stress [1,2].

In order to reduce the toxicity of chromate, dichromate and cadmium, some microorganisms eliminate these metals from the cytoplasm by using active transport efflux pumps [1,2]. Cadmium can also be sequestered within the cells by metal-chelating proteins, while chromate and dichromate are reduced to the less toxic and insoluble trivalent cation Cr(III) by specific NAD(P)H-dependent enzymes under aerobic conditions or in the electron transport chain of bacteria such as *Pseudomonas fluorescens* LB300 in anaerobic environments [4-9]. In addition, several enzymes work to counteract the deleterious effects of the oxidative stress induced following cell exposure to chromate, dichromate and cadmium.

*Caulobacter crescentus*, an oligotrophic free-living α-proteobacterium, is able to grow in polluted habitats [10-12]. Not surprisingly, its genome encodes some homologues of genes involved in heavy metal resistance. In a previous report, the set of genes responding to *Caulobacter* exposure to chromate, dichromate and cadmium was identified [12]. The main actions triggered in response to these metals are protection against oxidative stress (strong induction of superoxide dismutase, glutathione S-transferase, thioredoxin, glutaredoxins and DNA repair enzymes) and reduction of intracellular metal concentration (down-regulation of a sulfate transporter under chromate and dichromate stresses that could reduce nonspecific uptake of these oxyanions, and up-regulation of multiple efflux pumps that could play a key role in removing cadmium from the cytoplasm). However, the signal transduction and control processes involved in the bacterial response to these heavy metals are still poorly characterized.

The *C. crescentus* genome encodes 13 extracytoplasmic function (ECF) sigma factors [13]. Two of them, the paralogous σ^T^ and σ^U^, are involved in the response to various environmental stress conditions, including chromium and cadmium stresses [12,14]. Additionally, σ^E^ mediates a rapid transcriptional response to cadmium, organic hydroperoxide, singlet oxygen and UV-A [15]. In a previous report, σ^F^ was found to be required for bacterial survival under hydrogen peroxide stress in the stationary growth phase, but no σ^F^-mediated transcriptional response to hydrogen peroxide could be observed [16]. Thus, the involvement of σ^F^ in a transcriptional response to environmental stresses still needs to be characterized. The observation that genes CC2906, CC3255 and CC3257, previously found to be dependent on σ^F^[16], are induced following *C. crescentus* exposure to chromate, dichromate and cadmium [12] suggested to us that σ^F^ could be involved in the transcriptional response to these heavy metals.

In the present work, we demonstrate the involvement of σ^F^ in chromium and cadmium stress responses. We also identified the set of genes regulated by σ^F^ by using global transcriptome analysis and characterized the promoter region of these genes by 5´RACE experiments and β-galactosidase assays. Furthermore, we investigated the role of the protein encoded by the second gene in the *sigF* operon (CC3252), here named NrsF, and two conserved cysteine residues in this protein on the σ^F^-mediated response to heavy metals.

## Results

### σ^F^ is involved in chromium and cadmium responses in *C. crescentus*

**Figure 1:**
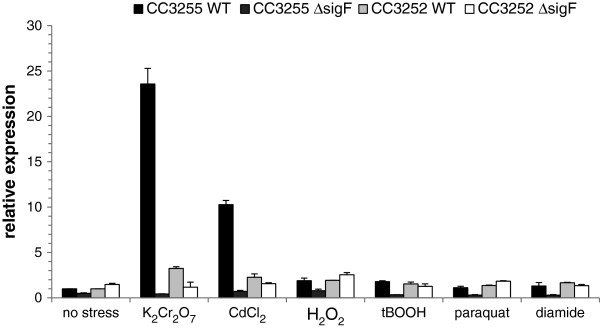
**Expression analysis of CC3255 and CC3252 under heavy metal stress.** qRT-PCR experiments were performed with total RNA extracted from exponentially growing cells immediately before and following exposure during 30 min to 55 μM potassium dichromate (K_2_Cr_2_O_7_), 55 μM cadmium chloride (CdCl_2_), 100 μM hydrogen peroxide (H_2_O_2_), 50 μM *tert*-butyl hydroperoxide (tBOOH), 100 μM paraquat or 50 μM diamide. Values represent the fold change in expression of CC3255 and CC3252 genes in parental strain NA1000 (WT) or the *sigF* mutant strain SG16 (Δ*sigF*), exposed or not to stress conditions, compared to the parental strain not exposed to stress. Results were normalized using gene CC0088 as the endogenous control, which was constitutively expressed under the conditions analyzed. Data are mean values of two independent experiments; bars represent the standard error. Statistical analysis is shown in Additional file 1: Table S4.

In order to verify a possible involvement of σ^F^ in the *C. crescentus* response to chromium and cadmium stresses, we monitored expression of CC3255, previously identified as a σ^F^-dependent gene, as well as CC3252, which is co-transcribed with *sigF* (CC3253), by quantitative RT-PCR. This analysis showed that CC3255 is significantly induced in parental cells following exposure to either dichromate or cadmium (Figure 1). In contrast, expression of CC3255 in a *sigF* deletion mutant strain exposed to dichromate or cadmium was found to be quite similar to that observed in the same strain under no stress condition (Figure 1). We could also observe significant induction of CC3252 in the presence of dichromate, though the induction was modest when compared to the increase in expression of CC3255 (Figure 1). On the other hand, no significant increase in CC3252 expression was found in *sigF* mutant cells following dichromate exposure (Figure 1). Taken together, these results confirm the involvement of σ^F^ in *C. crescentus* response to chromium and cadmium stresses and suggest that the operon *sigF*-CC3252 is not strongly auto-regulated under these conditions. To simplify our analyses and data presentation, we only show the expression of *sigF* and its target genes under dichromate stress in all subsequent experiments.

It is assumed that heavy metal ions cause oxidative stress inside cells [1,12,17]. This raises the possibility that induction of σ^F^-dependent genes by chromium and cadmium is a direct consequence of oxidative stress. To test this hypothesis, we stressed the parental and the *sigF* mutant strains with hydrogen peroxide, t-butyl hydroperoxide, paraquat (source of superoxide anion) or diamide (causes depletion of thiols). According to qRT-PCR experiments, expression levels of CC3255 and CC3252 were not increased more than twofold in the parental strain during these stress conditions (Figure 1). In agreement, transcript levels of CC3255 and CC3252 were also not influenced by any of these stressors in cells lacking *sigF*. Concentrations of hydrogen peroxide and t-butyl hydroperoxide used in our analyses were previously found to be sufficient to increase expression of other genes in *C. crescentus*[15,18]. Taken together, these data suggest that chromium and cadmium are able to induce the σ^F^ regulon in an oxidative stress independent manner.

### σ^F^ controls a small set of genes under chromium stress

**Figure 2:**
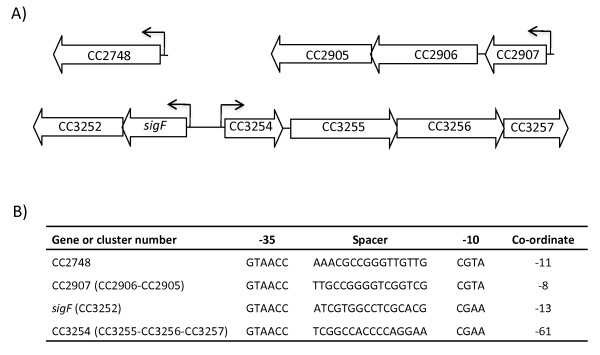
**σ**^**F**^**-dependent genes and promoters.A**. Genome organization of σ^F^-dependent genes. For each open reading frame, the locus name and orientation on chromosome are indicated. Predicted σ^F^-dependent promoters are shown by arrows. Organization of genes in operons was based on our transcriptome data and analyses of genomes presenting homologous of σ^F^-dependent genes. **B**. Table showing the putative −35 and −10 promoter elements of genes directly regulated by σ^F^. Promoter sequence motifs upstream from CC2907 and CC3254 were determined by 5´RACE experiments, while promoter elements of CC2748 were identified by a search for the σ^F^-binding sequence (GTAACC-N_16_-CGAA) in the region encompassing nucleotides −600 to +100 relative to the predicted translation start site (+1), allowing for two substitutions. The “dna pattern” tool of RSA website (http://rsat.ulb.ac.be/rsat) was used in this search. The coordinate represents the position of the 3’end nucleotide of the putative σ^F^-binding motif relative to the translation start site (+1). These sequences were compared to the promoter sequence located upstream of *sigF*, which was experimentally determined by primer extension [16]. Genes in parenthesis are proposed to be co-transcribed with the gene immediately downstream from the putative σ^F^-binding motif.

With the aim of identifying additional genes induced during stress conditions under the control of σ^F^, we compared the gene expression pattern of parental cells with that of a *sigF* mutant under dichromate stress, using microarray chips containing up to three different probes corresponding to the beginning of the coding region of each gene from *C. crescentus*. By considering these probes, we found only six genes down-regulated in *sigF* mutant cells relative to the parental strain (CC2748, CC2905, CC2906, CC3255, CC3256 and CC3257) (Table 1). Interestingly, close inspection of probes corresponding to the upstream region from CC2906 and CC3255 suggested that these regions are also down-regulated in *sigF* mutant cells when compared to the parental strain. The transcriptional start sites of the operons CC2906-CC2905 and CC3255-CC3256-CC3257 seem to be located quite distant from the translational start sites of CC2906 and CC3255 predicted by the TIGR annotation. Genome organization suggests that CC3254 is the first gene of the transcriptional unit CC3254-CC3255-CC3256-CC3257 (Figure 2A). According to the TIGR annotation, the deduced amino acid sequence of CC3254 displays an N-terminal extension of 57 amino acid residues not found in orthologous sequences. By excluding this extension, the most probable translational start site of CC3254 is at position +172 relative to the translational start site of CC3254 suggested by the TIGR annotation (Figure 2A). Thus, all probes designed to measure CC3254 expression in microarray chips correspond to a region upstream from the translational start site of CC3254 proposed here. However, probes corresponding to the upstream region of CC3255 cover the entire coding region of CC3254. Therefore, by considering these probes, we could include CC3254 as a σ^F^-dependent gene (Table 1). This is in accordance with the previous observation that the complete transcriptional unit CC3254-CC3255-CC3256-CC3257 is induced under chromium and cadmium stresses [1,12,17].

**Table 1:** **Expression analysis of σ**^**F**^**-dependent genes upon dichromate stress**

					**Microarray**^***f***^	**qRT-PCR**^***g***^		
**Gene number**^***a***^	**Length**^***b***^	**TM**^***c***^	**Domain**^***d***^	**Putative identification**^***e***^	**Δ*sigF*Cr/ WT Cr**	**WT Cr/ WT no stress**	**Δs*igF*Cr/Δ*sigF*no stress**	**Δ*sigF*Cr/WT Cr**
CC2748	313		Oxidored_molyb	sulfite oxidase subunit YedY	−2.097	4.654	2.500	−2.154
CC2905	261		DUF2063	protein of unknown function	−1.299	2.164	−0.481	−2.645
CC2906	289		DUF692	protein of unknown function	−2.917	3.358	0.967	−2.392
CC2907	105	1	DUF2282	predicted integral membrane protein	−2.386	NA	NA	NA
CC3252	214	6	DUF1109	negative regulator of σ^F^	NC	1.577	0.265	−1.312
CC3253	179		Sigma70_r2 Sigma70_r4	ECF sigma factor σ^F^	NC	NA	NA	NA
CC3254	93	1	DUF2282	predicted integral membrane protein	−4.904	NA	NA	NA
CC3255	280		DUF692	protein of unknown function	−4.783	4.697	−1.123	−5.820
CC3256	254		DUF2063	protein of unknown function	−3.311	NA	NA	NA
CC3257	150	2	DoxX	protein of DoxX family	−2.644	2.473	−2.879	−5.352

^*a*^ according to CMR (“*Comprehensive Microbial Resource*”) annotation of genome of CB15 strain.

^*b*^ referring to the number of amino acid of the deduced protein sequence. Protein length is according to CMR annotation or prediction from our analysis.

^c^ corresponding to the number of possible transmembrane (TM) helices in the mature protein. The number was determined by TMHMM tool.

^*d*^ according to a re-analysis of the deduced protein sequences by using Pfam and SMART tools to search for conserved domains.

^*e*^ sequences were compared with protein databases using Blastp.

^*f*^ microarray hybridization of RNA samples isolated from exponential phase cells exposed to 55 μM potassium dichromate (K_2_Cr_2_O_7_, denoted as Cr) for 30 min. Genes with *M* value of < −1.0 or > 1.0 were assumed as differentially expressed between strains analyzed. Values are the log_2_ ratio as mentioned. Results shown are the average of three independent biological experiments. WT and Δ*sigF* refer to the parental strain NA1000 and *sigF* deletion mutant, respectively. NC refers to no significant change in gene expression.

^*g*^ quantitative RT-PCR experiments performed with total RNA extracted from exponentially growing cells immediately before (no stress condition) and following exposure during 30 min to 55 μM potassium dichromate (K_2_Cr_2_O_7_, denoted as Cr). Results were normalized using gene CC0088 as the endogenous control, which was constitutively expressed under the conditions analyzed. Values are the log_2_ ratio as mentioned. Data are mean values of two independent experiments. WT and Δ*sigF* refer to the parental strain NA1000 and *sigF* deletion mutant, respectively. NA corresponds to genes not analyzed in qRT-PCR experiments.

The CC2907 gene is predicted to be transcribed divergently from CC2906-CC2905 in the chromosome of CB15 strain. However, the corresponding gene was not included during annotation of the more recent genome sequencing of *C. crescentus* (NA1000 strain). In the chromosome of NA1000, an open reading frame (CCNA_03001) was proposed to be located between genes CCNA_03000 (corresponding to CC2906) and CCNA_03002 (corresponding to CC2908). Nevertheless, CCNA_03001 appears to be co-transcribed with CCNA_03000 and CCNA_03002. In addition, we could observe co-occurrence of CCNA_03001 with other σ^F^-dependent genes. As the nucleotide sequence between CC2906 and CC2908 in CB15 strain is identical to the region between CCNA_03000 and CCNA_03002 of NA1000 strain, we conclude that CC2907 was incorrectly annotated in the genome of CB15 strain and this gene is the first one of the operon CC2907-CC2906-CC2905 (Figure 2A). As evaluated with probes corresponding to the upstream region of CC2906, the entire coding region of CC2907 is down-regulated in *sigF* mutant cells relative to the parental strain (Table 1). Therefore, the complete transcriptional unit CC2907-CC2906-CC2905 is controlled by σ^F^.

A thorough re-annotation of genes regulated by σ^F^ suggested that CC3257 codes for a putative membrane protein belonging to the DoxX family, whose members are involved in sulfur metabolism. The CC2748 gene, which encodes the putative sulfite oxidase subunit YedY, is another protein with a potential role in sulfur metabolism. All of the remaining σ^F^-dependent genes (CC2905, CC2906, CC2907, CC3254, CC3255 and CC3256) code for proteins with conserved domains of unknown functions. Interestingly, the pairs of genes CC2907 and CC3254, CC2906 and CC3255, as well as CC2905 and CC3256 are probable paralogous genes, with amino acid sequence identities of 36%, 43% and 23%, respectively. Therefore, it is possible that the gene products of both operons exert similar functions. No other gene in the genome of *C. crescentus* displays significant nucleotide sequence similarity to the above mentioned pairs of paralogous genes or to the functionally annotated genes CC2748 and CC3257.

Proteins encoded by CC2905 and CC3256 present a DUF2063 domain at their N-terminus. This domain was described to be a DNA-binding domain in NGO1945 from *Neisseria gonorrhoeae*[19]. NGO1945 is involved in the transcriptional regulation of *msrAB*, which codes for a methionine sulfoxide reductase [20]. However, in our microarray experiments, we could not observe differences in the expression of *msrA* homologs in *C. crescentus* (CC0994 and CC1039). Thus, we conclude that the role of NGO1945 in *N. gonorrhoeae* and CC2905 or CC3256 in *C. crescentus* is most likely different under these circumstances.

To confirm results obtained in transcriptome analysis, we investigated the expression levels of five genes supposedly dependent on σ^F^ (CC2748, CC2905, CC2906, CC3255 and CC3257) by qRT-PCR experiments. These analyses showed that expression of these selected genes under dichromate stress is more than twofold higher in the parental strain relative to the *sigF* deletion mutant (Table 1). Interestingly, induction of CC2748 expression in the presence of dichromate was only partially dependent on σ^F^ (Table 1), suggesting the involvement of an additional regulatory protein in the control of CC2748 expression under this stress condition. Taken together, these results confirm the data obtained in global transcriptional analysis.

### Promoter sequence motifs of CC2907 and CC3254 genes are highly similar to those of *sigF*

To identify putative σ^F^-dependent promoters upstream of CC2907 and CC3254 genes, we performed 5’RACE (rapid amplification of cDNA-ends) experiments using primers that hybridize in the beginning of the coding region of the corresponding genes. For these experiments, RNA samples from cells exposed to dichromate were used, as this stress condition leads to increased expression levels of CC2907 and CC3254. This approach led to the identification of a transcriptional start site (TSS) for CC2907 at position −7 relative to the translational start site +1 proposed here (Figure 2B). A TSS was also determined at position

−61 with respect to the translational start site of CC3254 predicted here (Figure 2B). As expected, no TSS could be observed when an additional 5´RACE experiment was performed using primers that hybridize to the beginning of the coding region of CC3254 proposed by the TIGR annotation. Together, these data confirmed our microarray data with respect to expression of the operons CC2907-CC2906-CC2905 and CC3254-CC3255-CC3256-CC3257.

The putative promoter sequences found for CC2907 and CC3254 were very similar to each other and also quite similar to the promoter sequence previously determined for *sigF*[16] (Figure 2B). Additionally, analyses of the region upstream of the translational start site +1 of CC2748 also revealed a putative σ^F^-dependent sequence (Figure 2B), suggesting a direct control of this gene by σ^F^. Accordingly, the putative σ^F^-dependent promoters reported here are highly similar to sequences found upstream from *sigF* homologs in other bacteria [21].

### Conserved sequences upstream of CC3254 and *sigF* are necessary for expression of these genes

**Figure 3:**
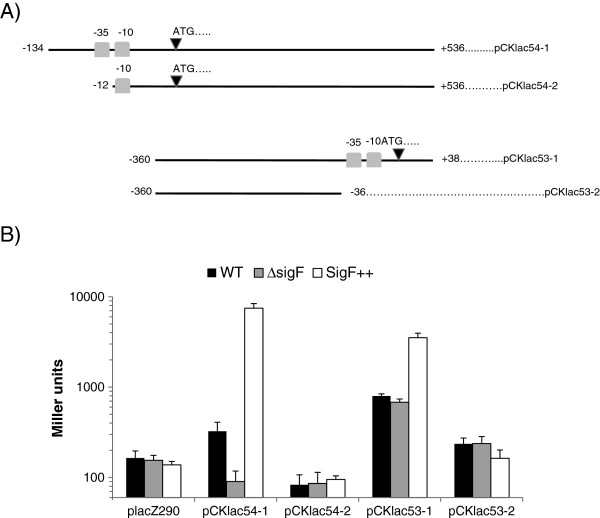
**Analysis of CC3254 and *sigF *promoter activity.A**. Illustration of the plasmid constructions used in β-galactosidase assays. Fragments containing the upstream region from CC3254 or *sigF* were obtained by PCR, sequenced and cloned into the plasmid placZ290 [46]. Light gray boxes represent the −35 and −10 promoter elements determined by 5´RACE experiment (CC3254) or by primer extension experiments (*sigF*) [16]. The black triangles correspond to the translation start sites. Numbers right and left indicate the position of 3’ and 5’ ends, respectively, relative to the transcription start site +1. **B**. β-galactosidase assays carried out with exponential growth phase cells from parental strain NA1000 (WT), *sigF* null mutant SG16 strain (Δ*sigF*) and *sigF* overexpressing cells (SigF^++^) containing the empty vector placZ290 or one of the different constructs with the upstream region of CC3254 or *sigF*. Data are mean values of three independent experiments; bars represent the standard error. Statistical analysis is shown in Additional file 1: Table S4.

To confirm the putative promoter sequence of the gene cluster CC3254-CC3255-CC3256-CC3257, transcriptional fusions containing a fragment encompassing the region upstream of the translational start site of CC3254 predicted in this work and the *lacZ* reporter gene (constructs pCKlac54-1 and pCKlac54-2) were created (Figure 3A). *Caulobacter* cells harboring these different constructs were used in β-galactosidase assays. When monitored in unstressed parental cells, a plasmid construction with the complete promoter sequence of the transcriptional unit CC3254-CC3255-CC3256-CC3257 (pCKlac54-1) resulted in higher β-galactosidase activity with respect to the empty vector placZ290 or to the construct lacking the −35 promoter element (pCKlac54-2) (Figure 3B). Only basal β-galactosidase activity was observed with any of the constructions in cells of the *sigF* null mutant strain (Figure 3B). These results confirmed the data from qRT-PCR and 5’RACE experiments. Analyses with dichromate stressed cells showed no significant increase in β-galactosidase activity in the parental strain (data not shown), probably due to the damage caused during dichromate stress to β-galactosidase in particular or to proteins in general.

As mentioned above, the promoter sequence of the operon CC3254-CC3255-CC3256-CC3257 is highly similar to that located upstream from *sigF*. To verify if *sigF* expression was also dependent on these putative promoter elements, we analyzed the upstream region of the *sigF* gene in β-galactosidase assays using two different plasmid constructs: pCKlac53-1 containing the promoter elements upstream from *sigF* and construct pCKlac53-2 that lacks the *sigF* promoter (Figure 3A). β-galactosidase activity measured in parental cells harboring the construct pCK53-2 (Figure 3B) was found to be quite similar to that observed in cells with the empty vector. On the other hand, higher β-galactosidase activity was observed in the parental strain carrying construct pCK53-1, which contains the complete *sigF* promoter sequence (Figure 3B). Cells from *sigF* mutant harboring the construct pCKlac53-1 presented β-galactosidase activity slightly lower than that observed in parental cells with the same construct, but still higher than that observed in cells harboring the construct pCK53-2 (Figure 3B). Altogether, these data indicate that the promoter sequence upstream from *sigF* is necessary for expression of the *sigF* operon, but in a manner that is not exclusively dependent on σ^F^. This observation suggests that another sigma factor could also be capable of recognizing the region upstream from *sigF*. Thus, we have investigated the effect of two other ECF sigma factors involved in oxidative and heavy metal stresses, σ^T^ and σ^E^, upon *sigF* promoter activity, but no significant decrease in β-galactosidase activity was observed in mutant strains Δ*sigT* and Δ*rpoE* when compared with parental cells, all harboring construct pCKlac53-1 (data not shown). Additionally, qRT-PCR experiments confirmed these results, as no change in transcription levels of the *sigF* gene was observed in a *sigT* or *rpoE* mutant (data not shown). These observations allowed us to rule out the participation of σ^T^ and σ^E^ in the control of *sigF* expression.

To further verify if the promoter region upstream of *sigF* is controlled by σ^F^, we overexpressed *sigF* in the parental strain from an additional plasmid-encoded copy of the gene under the control of a constitutive promoter (construct pCM30) and measured β-galactosidase activity in these cells harboring either pCKlac53-1 or pCKlac53-2. Overexpression of *sigF* in cells with the construct containing the complete *sigF* promoter (pCK53-1) led to an increase in β-galactosidase activity, whereas no difference was observed in cells harboring the promoterless construct pCKlac53-2 (Figure 3B). Similarly, higher β-galactosidase activity was observed in *sigF* overexpressing cells bearing the construct containing the promoter sequence motifs upstream from CC3254 (pCKlac54-1) when compared to the parental strain carrying the same construct or *sigF* overexpressing cells harboring the construct containing only the −10 motif of the promoter sequence of CC3254-CC3255-CC3256-CC3257 (pCKlac54-2) (Figure 3B). Therefore, these results confirm that specific and highly similar promoter sequence motifs found upstream from *sigF*-CC3252 and CC3254-CC3255-CC3256-CC3257 are required for the control of these transcriptional units by σ^F^.

### CC3252 negatively regulates σ^F^ regulon expression

**Figure 4:**
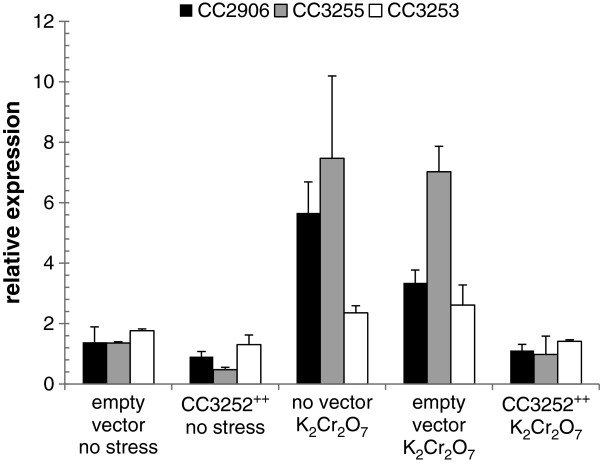
**Role of CC3252 on expression of CC2906, CC3255 and *sigF *genes.** Results shown are from qRT-PCR performed with total RNA extracted from exponential growth phase cells under control conditions (no stress) or stressed with potassium dichromate (K_2_Cr_2_O_7_). We analyzed the parental strain NA1000 without expression plasmid pJS14, NA1000 with the empty plasmid pJS14 and NA1000 with pJS14 containing CC3252 gene (CC3252^++^). Values represent the fold increase of CC2906, CC3255 and CC3253 (*sigF*) expression in the corresponding strain, exposed or not to the stress condition, compared with the parental strain NA1000 without pJS14 growing under control conditions. Results were normalized using gene CC0088 as the endogenous control, which was constitutively expressed in the samples analyzed. Data are mean values of two independent experiments; bars represent the standard error. Statistical analysis is shown in Additional file 1: Table S4.

The chromosomal organization of CC3252 and *sigF* in a putative operon suggests that CC3252 could be involved in the same regulatory pathway of σ^F^. To test the assumption that CC3252 could control σ^F^ activity, we monitored the expression of σ^F^-dependent genes in parental cells overexpressing CC3252 from a plasmid-encoded copy of the gene under the control of the constitutive *lacZ* promoter present in vector pJS14. For that, cells overexpressing CC3252 were stressed or not with dichromate and compared in qRT-PCR experiments with cells harboring the empty vector pJS14 or cells without this vector under the same conditions. According to qRT-PCR experiments, expression of genes CC2906 and CC3255 was slightly reduced in cells overexpressing CC3252 under no stress conditions, when compared to cells with the empty vector pJS14 or cells without the vector (Figure 4). However, induction of CC2906 and CC3255 expression under dichromate stress was clearly absent in CC3252 overproducing cells, when compared to cells not overexpressing CC3252 (Figure 4). No difference could be found in the expression levels of two control genes (CC1039 and CC0566) when we compared cells overexpressing CC3252 or not (data not shown). This observation rules out a possible nonspecific effect due to overproduction of the protein. Taken together, these data indicate that CC3252, here denominated *nrsF*, acts as a negative regulator of σ^F^ function in *C. crescentus*. Upon dichromate stress, *sigF expression* was also slightly down-regulated in CC3252 overexpressing cells, whereas under control conditions *sigF* expression was practically unchanged. This once again favors the hypothesis that *sigF* is not strongly auto-regulated.

A further attempt to investigate the role of *nrsF* as a possible negative regulator of σ^F^ function was carried out by trying to construct a null mutant strain in gene *nrsF*. However, it was not possible to construct a mutant strain by deleting *nrsF* in the parental strain (data not shown). On the other hand, *nrsF* could be deleted in the absence of a functional copy of *sigF* (data not shown), suggesting that high σ^F^ activity is apparently responsible for the failure of disrupting *nrsF* in cells with functional *sigF*.

**Figure 5:**
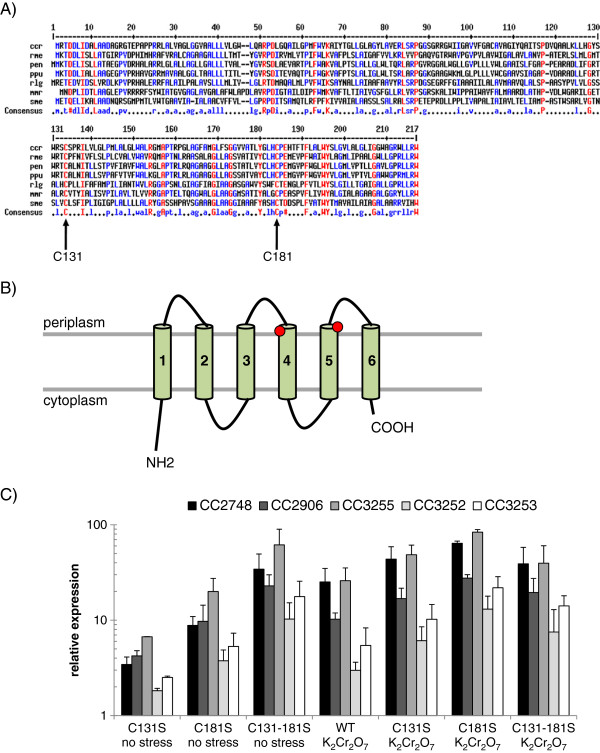
**Role of the conserved cysteines C131 and C181 of CC3252 upon expression of σ**^**F**^**-dependent genes.A**. The deduced protein sequences of orthologs of CC3252 obtained from *Cupriavidus metallidurans* (rme), *Pseudomonas entomophila* (pen), *Pseudomonas putida* (ppu), *Rhizobium leguminosarum* (rlg), *Maricaulis maris* (mmr) and *Sinorhizobium meliloti* were compared with CC3252 deduced protein sequence of *Caulobacter crescentus* (ccr) using MultiAlign [47]. Arrows assign the conserved cysteines C131 and C181 of *C. crescentus* in all orthologs. **B**. Illustration of the putative topology of the deduced protein sequence encoded by CC3252 on the inner membrane. The six transmembrane segments were predicted using SMART [48] and are indicated by green cylinders. Conserved cysteine residues and denoted as red circles. **C**. qRT-PCR was performed using total RNA extracted from exponential growth phase cells from parental strain NA1000 and mutant strains SG22 (C131S), SG23 (C181S) and SG24 (C131S-181S) cultured under unstressed condition (no stress) or following exposure to 55 μM potassium dichromate (K_2_Cr_2_O_7_) for 30 min. Values represent the fold increase of CC2748, CC2906, CC3255, CC3252 and CC3253 (*sigF*) expression in the corresponding strains exposed or not to the stress condition compared with that of the parental strain NA1000 growing under no stress conditions. Results were normalized using gene CC0088 as the endogenous control, which was constitutively expressed in the samples analyzed. Data are mean values of two independent experiments; bars represent the standard error. Statistical analysis is shown in Additional file 1: Table S4.

The putative protein encoded by *nrsF* is composed of six putative transmembrane segments separated by five short linkers (6 to 19 amino acid residues) and an N-terminal segment of 25 residues (Figure 5B). Alignment of the deduced amino acid sequence of CC3252 with its orthologs from other bacteria (*Cupriavidus metallidurans, Pseudomonas entomophila, Pseudomonas putida, Rhizobium leguminosarum, Maricaulis maris* and *Sinorhizobium meliloti*) revealed two highly conserved cysteine residues (Figure 5A). The cysteine residues of the *Caulobacter* protein (positions 131 and 181) are probably directed into the periplasmic space (Figure 5B), which favors their putative role in the signal transduction process leading to the liberation of σ^F^ from NrsF inhibition. Substitution of the conserved cysteines by serine led to two single mutants (SG22, C131S; SG23, C181S) and a double mutant (SG24, C131S-C181S). Even under unstressed conditions, all σ^F^-regulated genes analyzed in qRT-PCR experiments, including *sigF* and CC3252, were up-regulated in the single mutant strains when compared to the parental strain (Figure 5C). The substitution of both cysteines by serine in NrsF resulted in the highest expression levels of the genes analyzed (Figure 5C). Under stress conditions, both single substitution mutant strains showed an additional induction of σ^F^-dependent genes, whereas no differences were observed for the double substitution mutant strain, compared to unstressed conditions (Figure 5C). Therefore, these results show that substitution of one or both of the conserved cysteines (C131 and C181) in the protein encoded by *nrsF* affects the ability of this protein in maintaining expression of σ^F^-dependent genes at basal levels, further indicating the negative role of *nrsF* in the control of σ^F^ activity.

### σ^F^ is released into the cytoplasm during chromium stress and in cells carrying point mutations in conserved cysteines of NrsF

**Figure 6:**
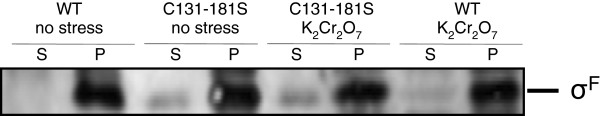
**Subcellular localization of σ**^**F**^**.** Immunoblot assays performed with membrane and soluble fractions obtained from parental strain NA1000 (WT) and a CC3252 mutant with both cysteine residues C131 and C181 replaced for serine (C131-181S). Aliquots were taken immediately before or after cells were treated with 55μM potassium dichromate (K_2_Cr_2_O_7_) for 30min. Membrane and soluble fractions were obtained as described in Methods. Blots were developed using anti-σ^F^antiserum and fluorescent CF680 Goat Anti-Rabbit IgG. σ^F^ is shown by an arrow.

The presence of six putative transmembrane segments in the protein coded by *nrsF* would imply that σ^F^ is sequestered to the inner membrane of *Caulobacter* cells. However, at least a portion of this sigma factor would be expected to be released into the cytoplasm following chromium and cadmium exposure. To investigate this assumption, we monitored σ^F^ levels in the membrane and soluble fractions of *Caulobacter* cell extracts by Western blot analysis (Figure 6). When extracts from parental cells under no stress condition were analyzed, σ^F^ was only detected in the membrane fraction. Although the majority of σ^F^ was still observed in the membrane fraction of extracts from parental cells exposed to dichromate, a significant portion of the sigma factor could also be detected in the soluble fraction. Additionally, we found that substitution of both conserved cysteine residues of NrsF also led to a significant accumulation of σ^F^ in the soluble fraction, even when cells were not exposed to dichromate. In contrast, the membrane-bound FtsH protease was only detected in the membrane fraction of both strains analyzed (not shown). Taken together, these results showed that cells displaying increased expression of σ^F^-dependent genes accumulate this sigma factor in the cytoplasm.

### Neither σ^F^ nor σ^F^-dependent genes CC2906 and CC3255 are essential for *Caulobacter* resistance to metal stress

To investigate the requirement of *sigF* for resistance of *C. crescentus* cells to dichromate or cadmium, the sensitivity of the parental strain and the *sigF* deletion mutant to exposure to these metals was monitored. Both strains displayed similar sensitivity profile to dichromate or cadmium (data not shown), suggesting that *sigF* is not essential for bacterial survival under this stress condition. As the deduced protein sequences of CC2906 and CC3255 are highly similar, we constructed a single deletion mutant strain in each gene (SG19 and SG20) as well as a double mutant (SG21) and tested the resistance of these strains to the metal stresses. Similar to what was found for the *sigF* deletion mutant, no increased sensitivity was observed for these mutant strains following exposure to either dichromate or cadmium, when compared to parental cells (data not shown). Together, these data suggest that σ^F^-mediated transcriptional response to chromium or cadmium is not essential for survival of *C. crescentus* to exposure to these metal ions.

## Discussion

In this report, we clearly show that *C. crescentus* σ^F^ is involved in the transcriptional response to chromium and cadmium in an oxidative stress independent manner. Transcriptome analysis of cells under dichromate stress revealed that σ^F^ controls a small regulon comprised of eight genes, which are distributed in three transcriptional units. Although a conserved domain was predicted for the deduced protein sequence of all σ^F^-dependent genes, only two of these sequences could be assigned to a possible function. The protein encoded by CC2748 belongs to the group of sulfite oxidases, which catalyze the oxidation of the toxic and very reactive sulfite to the inert sulfate anion [22]. The product of CC3257 is a member of the DoxX family. Although nothing is actually known about the physiological role of bacterial proteins belonging to this family, the archaeal counterparts are involved in the elemental sulfur oxidation pathway [23,24]. Therefore, both σ^F^-dependent genes with a putative assigned function appear to play a role in sulfate acquisition by cells. Interestingly, Hu et al. (2005) found a strong down-regulation of a *Caulobacter* sulfate ABC transport system under chromate and dichromate exposure. While this detoxification strategy apparently contributes to decrease the concentration of chromate and dichromate in the cells [4], sulfate uptake from the extracellular environment might be significantly affected. Alternative sources such as degradation of sulfur-containing amino-acids [25] and organosulfonate metabolism [26] can be used to counteract this sulfur uptake limitation [1,27-29]. It is therefore conceivable that induction of CC2748 and CC3257 could supply cells with sulfate. This is consistent with the observation that in *Arthrobacter sp*. strain FB24 and *Pseudomonas putida*, chromate exposure also results in increased levels of proteins potentially involved in reversing the effects of cellular sulfur limitation, such as transporters of alternative sulfur sources [27,28].

Curiously, none of the most representative functional categories up-regulated under chromate, dichromate or cadmium exposure (protection against oxidative stress and reduction of intracellular metal concentration) were found to be controlled by σ^F^, indicating that additional molecular systems are engaged in *C. crescentus* response to these metals. In fact, we previously reported the involvement of the paralogous sigma factors σ^T^ and σ^U^ in the control of response to chromium and cadmium [14,15,30] and σ^E^ in response to cadmium [14,15,30]. The observation that σ^F^, σ^E^ and σ^T^/σ^U^ regulate distinct sets of genes indicates that each of these sigma factors make a different contribution to the *C. crescentus* response to metal stress. Together, σ^F^, σ^E^, σ^T^ and σ^U^ are responsible for the induction of 20% of the genes previously found to be up-regulated under cadmium stress and σ^F^, σ^T^ and σ^U^ control the expression of about 12% of genes induced following *Caulobacter* exposure to chromate or dichromate (Additional file 1: Table S1). Therefore, transcriptional regulators other than σ^F^, σ^E^, σ^T^ and σ^U^ appear to be involved in the response to chromate, dichromate and cadmium. The existence of several molecular systems contributing to the transcriptional response to metal stresses could explain why the absence of *sigF,* CC2906 or CC3255 does not decrease the viability of *Caulobacter* cells under dichromate or cadmium stresses. In agreement, we previously reported that σ^E^ elicits a rapid response to cadmium, but cells lacking *rpoE* are not impaired in survival to this stress condition [14,15,30].

Interestingly, *sigF* is not highly induced under either chromium or cadmium stress, different from what was observed for other ECF sigma factor genes such as *rpoE* and *sigT* in *C. crescentus*[14,15,30] and *rpoJ*, *rpoK*, *rpoI*, *cnrH* and *rpoQ* in *C. metallidurans*[31]. This indicates that *sigF* is obviously not strongly auto-regulated under heavy metal stress conditions. Although the experimentally determined promoter sequences of *sigF* and CC3254 are highly similar to each other, promoter activity analyses supported our observation that CC3254 is solely regulated by σ^F^, while the *sigF* operon is transcribed under the control of σ^F^ and a still unknown transcriptional regulator. Interestingly, both σ^F^ and the additional regulator depend on sequences located from −37 to +37 relative to the transcriptional start site (+1) of *sigF*. An apparent competition between these proteins might be the reason why *sigF* promoter activity is less responsive to high levels of σ^F^ when compared to the CC3254 promoter, which is solely controlled by σ^F^. The existence of a second regulator of the *sigF* operon would be important to maintain a certain basal level of σ^F^ and consequently to allow a rapid response when cells experience environments contaminated with heavy metals. In the literature, one can find various examples of ECF sigma factor genes dependent on a second ECF sigma factor [32,33]. In the present study, we could exclude *Caulobacter rpoE* and *sigT* as possible regulators of σ^F^, since no difference in *sigF* expression was observed in the absence of either one of these ECF sigma factor genes.

In most cases, the activity of ECF sigma factors is modulated by a cognate anti-sigma factor [34-36]. Here, we showed that the second gene (CC3252) in the *sigF* operon acts as a negative regulator of σ^F^ function, as overexpression of the putative membrane protein encoded by CC3252 abolishes the transcriptional activation of *sigF* and its regulon under dichromate stress. Thus, CC3252 was here denominated *nrsF*. An interesting question about the nature of σ^F^ inhibition came from the observation that most of the protein encoded by *nrsF* is predicted to lie in the inner membrane of the bacterium: six transmembrane helices separated by five linkers ranging from 6 to 19 amino acid residues and an N-terminal segment of 25 residues. Usually, anti-sigma factors bind their cognate sigma factor through an extensive surface interaction, in which a domain of the first protein is sandwiched between domains σ_2_ and σ_4_ of the sigma factor [37]. It is possible that several of the linkers of NrsF contact σ^F^, resulting in a more stable interaction surface. However, we cannot discard the presence of a third component in this system able to directly bind both σ^F^ and NrsF and transduce the signal leading to activation of the sigma factor, to compensate this apparent lack of sufficient cytoplasmic segments in NrsF to contact σ^F^. Attempts to obtain soluble recombinant full-length NrsF failed, probably because the protein cannot correctly fold in the absence of the hydrophobic environment found in the membrane compartment of bacterial cells. Therefore, it was not possible to test whether the recombinant protein encoded by *nrsF* directly binds σ^F^.

As previously observed for other ECF sigma factors of *C. crescentus*[14,15,30], we were not able to delete *nrsF*, probably due to the toxic effect of high levels of σ^F^ under no stress conditions. However, we could isolate strains in which one or both of the conserved cysteine residues of NrsF were replaced for serine. As suggested by Western blot analysis, isolation of these point mutation strains was possible probably because most of σ^F^ molecules are still directly or indirectly sequestered in an inactive state to the inner membrane by NrsF. Substitution of the conserved cysteines might have caused structural changes in NrsF and hence resulting in a lower capacity to bind σ^F^. In fact, σ^F^ was found to accumulate in the soluble fraction of cells expressing NrsF mutated in both cysteine residues even when cells were cultured under unstressed conditions. The presence of σ^F^ in the soluble fraction was also detected following treatment of parental cells with dichromate. Therefore, we could observe accumulation of σ^F^ in the soluble fraction in situations in which lower affinity of NrsF for σ^F^ is expected. Interestingly, two conserved cysteine residues in ChrR, the anti-sigma factor of *Caulobacter* σ^E^, were also shown to be important for the response to cadmium mediated by that sigma factor [14,15,30]. Furthermore, the sensor histidine kinase PhyK, involved in the control of the anti-anti-sigma factor PhyR of *Caulobacter* σ^T^, which as mentioned above responds to dichromate and cadmium, also presents a conserved cysteine that is important to PhyK activity [14,15,30]. Thus, cysteines in the probable sensor proteins (NrsF, ChrR and PhyK) of ECF sigma factor mediated systems seem to play a key role in triggering the response to heavy metal stress in *C. crescentus*.

Based on the fact that dichromate and cadmium are able to directly bind thiol groups [2,38], it is conceivable that these metals could disrupt contacts mediated by the conserved cysteines of NrsF, leading to changes in its conformation similar to those expected in the mutant proteins with one or both of the cysteine residues substituted. However, activation of σ^F^ might also be caused by direct interaction of chromate, dichromate and cadmium with other amino acid residues in NrsF or even with another yet unknown sensory component of the system. The finding that single substitutions of the conserved cysteine residues still allows for induction of σ^F^-dependent genes ruled out the formation of an intramolecular bond between Cys131 and Cys181 residues under stress conditions. Nevertheless, we could not discard the possibility that NrsF functions as a dimer/multimer using intermolecular bonds for sensing the metals in the extracytoplasmic environment.

## Conclusion

This report deals with the role and regulation of *C. crescentus* σ^F^ under stress conditions and provides new interesting information about this conserved but still poorly characterized ECF sigma factor: i) σ^F^-dependent genes are induced in the presence of heavy metals in a manner independent of oxidative and disulfide stress; ii) σ^F^ directly controls a small regulon including genes involved in sulfur metabolism and iii) σ^F^ is negatively regulated by a putative membrane bound protein, here named NrsF, which contains two conserved cysteine residues that are important for its function and are located in the periplasmic portion of the protein.

## Methods

### Bacterial strains and growth conditions

The strains and plasmids used in this study are described in Additional file 1: Table S2. *C. crescentus* strains were cultured at 30°C in M2 minimal salts medium plus glucose [39]. When appropriate, the growth medium was supplemented with chloramphenicol (1 μg ml^-1^), kanamycin (10 μg ml^-1^) or tetracycline (2 μg ml^-1^). Plasmids were propagated in *Escherichia coli* strain DH5α (Invitrogen) and mobilized into *C. crescentus* by bacterial conjugation using *E. coli* strain S17-1 [40]. *E. coli* strains were grown at 37°C in LB broth [41].

### Deletion of genes CC2906 and CC3255 in *C. crescentus*

Single mutant strains for CC2906 (SG20) and CC3255 (SG19) were obtained by an in-frame deletion in the coding region of these genes. For that, two fragments flanking the regions to be deleted were amplified by PCR (a complete list of primers used in this study is in Additional file 1: Table S3) and subcloned into pNPTS138 [42]. Constructs into pNPTS138 were transferred to *C. crescentus* strain NA1000 [43] by conjugation with *E. coli* S17-1 and the deletion of the wild-type copy of the gene in the NA1000 background was achieved by two homologous recombination events. Mutant strains were isolated by screening colonies by PCR and DNA sequencing. For the construction of a double mutant strain (SG21), the single mutant strain SG20 was used for the two homologous recombination events of the CC3255 deletion.

### Construction of point mutations in CC3252 and overexpression of CC3252 in *C. crescentus*

Codons for the conserved cysteine residues of the protein encoded by CC3252 (C131 and C181) were replaced for a codon corresponding to serine by overlapping PCR with a pair of complementary primers (Additional file 1: Table S3) designed for each substitution. Each part of CC3252 was amplified separately by PCR using one of each complementary primer set and a primer hybridizing upstream or downstream from CC3252. The partially complementary PCR products were used together as templates in a second amplification reaction with the primers hybridizing upstream and downstream from CC3252. The amplicons obtained were cloned into pGEM-T (Promega) and sequenced. The inserts were excised from vectors and subcloned into pNPTS138. Constructs into pNPTS138 were transferred to *C. crescentus* strain NA1000 [43] by conjugation with *E. coli* S17-1 and replacement of the wild-type copy of the gene for the corresponding mutated copy in the NA1000 background was achieved by two homologous recombination events. Mutant strains were isolated by screening colonies by PCR and DNA sequencing. To overexpress CC3252 in *C. crescentus* cells, a fragment corresponding to the coding region of the gene was first amplified by PCR. This fragment was excised from the vector and ligated into pJS14. The construct was introduced into *C. crescentus* NA1000 by conjugation with *E. coli* S17-1.

### RNA extraction

For quantitative real time-PCR (qRT-PCR) analysis, cultures of different *C. crescentus* strains were grown to exponential phase (OD_600_ 0.5), submitted for 30 minutes to stress (55 μM dichromate, 55 μM cadmium, 100–500 μM hydrogen peroxide, 50–200 μM t-butyl hydroperoxide, 100–500 μM paraquat or 50–200 μM diamide) or kept under no stress conditions and cells (four aliquots of 2 ml from each treatment) were collected by centrifugation in a microcentrifuge for 1 min. For microarray experiments, total RNA was extracted from the parental NA1000 and the *sigF* mutant strain SG16 at the exponential growth phase exposed to 55 μM dichromate for 30 min.

The cell pellets were suspended in 1 ml of Trizol Reagent (Invitrogen), and after the extraction procedure according to manufacturer’s instructions, the integrity of the RNA was checked by agarose gel electrophoresis and tested for the absence of DNA contamination by PCR.

### Quantitative real-time PCR

Reverse transcription for qRT-PCR was performed using 5 μg of total RNA, 200 U of Superscript III reverse transcriptase (Invitrogen) and 500 ng of random primer, following manufacturer’s instructions. Quantitative PCR amplification of the resulting cDNA was performed with Platinum SYBR Green (Applied Biosystems) and gene-specific primers (see Additional file 1: Table S3). These primers were designed using the Primer Express software (Applied Biosystems). Results were normalized using CC0088 gene as the endogenous control, which was previously used [15,30] and shown to be constant in the samples analyzed. Relative expression levels were calculated using the 2^-ΔΔCT^ method [44].

### 5’RACE

RNA 5’ ends of genes of interest were determined using the 3^′^/5^′^RACE kit (Roche). For that, the RNA was reverse transcribed using a gene-specific primer (Additional file 1: Table S3), purified and poly(dA) tailed at their 3^′^ends. The resulting cDNA was amplified by PCR using the forward poly(dT)-anchor primer provided by the kit to anneal at the poly(dA) tail and a second gene-specific primer. The PCR products were used in a second PCR reaction using a primer complementary to the poly(dT)-anchor primer and a distinct gene-specific nested primer. PCR products were ligated into the pGEM-T vector (Promega) and several distinct clones were sequenced.

### Microarray analysis

Three distinct biological RNA samples isolated from each strain analyzed were reverse transcribed and labeled using the FairPlay III Microarray Labeling system (Agilent). Briefly, the cDNA was synthesized from total RNA (20 μg) in the presence of amino allyl modified dUTP and random primer. After purification, the resulting amino-modified cDNA was chemically labeled by incorporation of the dyes Alexa Fluor 555 (Cy3) or Alexa Fluor 647 (Cy5). Labeled cDNAs were combined, mixed with Agilent hybridization buffer, and competitively hybridized to custom-designed Agilent microarrays according to the manufacturer’s instructions (Agilent). Data extraction and normalization was performed using Agilent Feature Extraction Software 9.5.3.1 (Agilent). The custom-designed arrays contain 9–11 probes covering a region around the translational start site (−300 to +200 relative to the translational start site +1) of each gene. Only those probes downstream of the translational start site were considered for estimating the fold change in gene expression. Ratios obtained for probes corresponding to the same gene were averaged and genes showing a ratio log2 (mutant/parental) < −1 or log2 (mutant/parental) > 1 in all three biological replicates were considered as differentially expressed between the strains analyzed. Complete microarray dataset was deposited in GEO (GSE 32406).

### Cell fractionation and Western blot analysis

Protein extracts were obtained from cultures of parental strain NA1000 and a CC3252 mutant with both C131 and C181 replaced for serine before and after treatment with 55 μM dichromate for 30 min. Cells were cultured until OD_600_ 0.5, harvest by centrifugation and washed once with 0.2 M Tris–HCl pH 8.0. Cells were then resuspended in 1 ml 60 mM Tris–HCl pH 8.0, 0.2 M sucrose, 0.2 mM EDTA, 200 μg ml^-1^ of lysozyme and incubated for 10 min at room temperature. After brief sonication (three 10 s pulses), cell debris were removed and the supernatant was centrifuged at 150,000 x g for 1 h. The pellet was washed once with 60 mM Tris–HCl pH 8.0 and resuspended in 1 ml 60 mM Tris–HCl pH 8.0, 0.2 M sucrose, 0.2 mM EDTA. Equal amounts of total protein (20 μg) were resolved through SDS-PAGE and transferred to nitrocelulose membrane, as previously described [45]. Membranes were incubated overnight at 4°C with anti-σ^F^ (1:500) [16] or anti-FtsH (1:2000) (kindly provided by T. Ogura, Kumamoto University, Japan) antibody in 10 mM Tris–HCl pH 8.0 containing 150 mM NaCl, 0.02% Tween 20, and 0.03% Triton X-100. The blots were developed using fluorescent CF680 Goat Anti-Rabbit IgG (1:10000- Uniscience) and imaged using Odyssey Imager- LI-COR (Biosciences).

### Promoter activity assay

β-galactosidase assays were carried out with cells carrying a CC3255-*lacZ* transcription fusion (pCKlac54-1 or pCKlac54-2) or a *sigF-lacZ* transcription fusion (pCKlac53-1 or pCKlac53-2). For that, cells were cultured to exponential phase, harvested and used for the enzymatic assay. The empty plasmid placZ290 [46] was used as the control in the experiments. β-galactosidase activity was measured as previously described [41]. All experiments were performed in duplicates and repeated on three different occasions.

### Stress sensitivity tests

Exponentially growing cells were exposed to 55 μM dichromate or kept under unstressed conditions. To measure cell viability, aliquots were removed, serially diluted and plated on M2 minimal medium for counting colony-forming units.

### Sequence alignment and structure prediction

Sequence comparison of orthologs of CC3252 was carried out using MultiAlign [47]. The transmembrane segments of CC3252 were predicted using SMART [48].

## Competing interests

The authors declare that they have no competing interests.

## Authors’ contribution

CK, RFL and SLG planned the experiments; CK, RFL and GMA conducted the experiments; CK and RFL analyzed as well as interpreted the data. CK, RFL and SLG prepared the manuscript. All authors read and approved the final manuscript.

## Supplementary Material

Additional file 1 **Table S1.** Genes induced by heavy metals and their potential controlling ECF sigma factors. **Table S2.** Strains and plasmids. **Table S3.** List of primers. **Table S4.** Statistical analysis of the data shown in the figures. Click here for file
