# Supplementary material for: Extracytoplasmic function (ECF) sigma factor σF is involved in Caulobacter crescentus response to heavy metal stress
Source: BMC Microbiol. 2012 Sep 18;12:210. doi: 10.1186/1471-2180-12-210 (PMC3511200; doi:10.1186/1471-2180-12-210)
Supplement: Additional file 1 — Table S1. Genes induced by heavy metals and their potential controlling ECF sigma factors. Table S2. Strains and plasmids. Table S3. List of primers. Table S4. Statistical analysis of the data shown in the figures. [file 1471-2180-12-210-S1.pdf]

Table S1. Genes induced by heavy metals and their potential controlling ECF sigma factors

gene	cadmium	chromate	dichromate
**CC0201**	σ^T/σ^U	σ^T/σ^U	σ^T/σ^U
**CC0280**	σ^T/σ^U	σ^T/σ^U	σ^T/σ^U
**CC0351**	σ^E		
**CC0419**	σ^E		
**CC0459**	σ^E		
**CC0646**	σ^E		
**CC0647**	σ^E		
**CC0648**	σ^E		
**CC0747**		σ^T/σ^U	σ^T/σ^U
**CC0956**	σ^T	σ^T	σ^T
**CC1039**	σ^E		
**CC1177**	σ^T	σ^T	σ^T
**CC1178**	σ^T/σ^U	σ^T/σ^U	σ^T/σ^U
**CC1179**	σ^T/σ^E	σ^T	σ^T
**CC1356**		σ^T/σ^U	σ^T/σ^U
**CC1427**	σ^T/σ^E	σ^T	σ^T
**CC1428**	σ^E		
**CC1452**		σ^T	σ^T
**CC1532**	σ^T/σ^U	σ^T/σ^U	σ^T/σ^U
**CC1958**		σ^T	
**CC2258**	σ^E		
**CC2498**		σ^T	
**CC2533**			σ^T

**CC2549**	σ^T	σ^T	σ^T
**CC2600**	σ^E		
**CC2748**	σ^E / $\boldsymbol{\sigma^F}$	$\boldsymbol{\sigma^F}$	$\boldsymbol{\sigma^F}$
**CC2872**	σ^T		
**CC2873**	σ^T/σ^U		
**CC2883**	σ^T/σ^U	σ^T/σ^U	σ^T/σ^U
**CC2906**		$\boldsymbol{\sigma^F}$	$\boldsymbol{\sigma^F}$
**CC3225**		σ^T/σ^U	σ^T/σ^U
**CC3254**	$\boldsymbol{\sigma^F}$	$\boldsymbol{\sigma^F}$	$\boldsymbol{\sigma^F}$
**CC3255**	$\boldsymbol{\sigma^F}$	$\boldsymbol{\sigma^F}$	$\boldsymbol{\sigma^F}$
**CC3256**	$\boldsymbol{\sigma^F}$	$\boldsymbol{\sigma^F}$	$\boldsymbol{\sigma^F}$
**CC3257**	$\boldsymbol{\sigma^F}$	$\boldsymbol{\sigma^F}$	$\boldsymbol{\sigma^F}$
**CC3260**		σ^T	σ^T
**CC3466**		σ^T/σ^U	σ^T/σ^U
**CC3473**	σ^T	σ^T	σ^T
**CC3475**		σ^T	σ^T
**CC3476**	σ^T	σ^T	σ^T
**CC3477**		σ^T	
**CC3554**		σ^T	σ^T
**CC3558**		σ^T	
**CC3559**		σ^T	σ^T
**CC3758**			σ^T

Genes identified in the whole-genome transcriptional analysis of heavy metal stresses (Hu *et al.*, 2005) and in transcriptome analyses carried out to identify genes controlled by σ^E (Lourenco & Gomes, 2009), σ^T (Lourenco *et al.*, 2011), σ^U (Lourenco *et al.*, 2011), and $\boldsymbol{\sigma^F}$ (present work) are displayed.

Table S2. Strains and plasmids

Strain or Plasmid	Description	Source or reference
Strains		
E.coli		
DH5α	Δ(*lacZYA-argF*) *U169 deoR recA1 endA1 hsdR17 phoA sup144 thi-1 gyrA96 relA1* (ϕ80 *lacZDM15*)	Invitrogen
S17-1	*F⁻ lambda thi pro recA hsdR⁻ hsdM⁺ RP4* derivative integrated into chromosome with *Tet::Mu; Km::Tn7*	(Simon, 1983)
C. crescentus		
NA1000	Holdfast mutant derivative of wild-type CB15	(Evinger & Agabian, 1977)
SG16	*sigF* in-frame deletion mutant	(Alvarez-Martinez *et al.*, 2006)
SG19	CC3255 in-frame deletion mutant	This work
SG20	CC2906 in-frame deletion mutant	This work
SG21	CC2906/CC3255 in-frame double deletion mutant	This work
SG22	Substitution of cysteine 131 (C131) to serine in the gene CC3252	This work
SG23	Substitution of cysteine 181 (C181) to serine in the gene CC3252	This work
SG24	Substitution of cysteine 131 (C131) and cysteine 181 (C181) to serine in the gene CC3252	This work
Plasmids		
pGEM-T	Cloning vector; Amp^r	Promega
pNPTS138	Derivative of pNPTS129 containing *sacB, nptI, oriT* and pLITMUS38 polylinker; Kan^r	M.R.K. Alley
pCK2	pNPTS138 with a 1039 bp fragment upstream of CC3255 and a 1039 bp fragment downstream of CC3255 for the deletion of CC3255	This work
pCK3	pNPTS138 with a 1012 bp fragment upstream of 2906 and a 978	This work

	bp fragment downstream of CC2906 for the deletion of CC2906	
pCK4	pNPTS138 with a 3059 bp fragment containing CC3252 with substitution of the codon corresponding to cysteine 131 to a codon for serine	This work
pCK5	pNPTS138 with a 3059 bp fragment containing CC3252 with substitution of the codon corresponding to cysteine 181 to a codon for serine	This work
pCK6	pNPTS138 with a 3059 bp fragment containing CC3252 with substitution of the codons corresponding to cysteine residues 131 and 181 to a codon for serine	This work
pJS14	Vector for constitutive expression of genes in *C. crescentus* from p*lacZ* promoter, medium copy number (10–20 copies per cell); Cm^r *oriT*	(Kovach *et al.*, 1995)
pCM3	pJS14 with transcriptional fusion to CC3252 gene	This work
pCM30	pJS14 with transcriptional fusion to *sigF*	(Alvarez-Martinez *et al.*, 2006)
placZ290	pK2-based vector for transcriptional fusions to the *lacZ;* Tet^r	(Gober & Shapiro, 1992)
pCKlac54-1	placZ290 with transcriptional *lacZ* fusion to CC3254 promoter region (from -134 to +536 relative to the transcriptional start site +1)	This work
pCKlac54-2	placZ290 with transcriptional *lacZ* fusion to CC3254 upstream region (from -12 to +536 relative to the transcriptional start site +1)	This work
pCKlac53-1	placZ290 with transcriptional *lacZ* fusion to *sigF* promoter region (from -360 to +38 relative to the transcriptional start site +1)	This work
pCKlac53-2	placZ290 with transcriptional *lacZ* fusion to *sigF* promoter region (from -360 to -36 relative to the transcriptional start site +1)	This work

Table S3. List of primers

Primer name	Sequence (5´-3´)	Usage
3254forHindIII	AATGAAGCTTCCCGAGCGCCTGCCTCGGC	For PCR fragment upstream of CC3255 (for the construction of SG19)
3254revEcoRI	ATGAATTCGTGCTGCGATTTGAGGCCAAG	For PCR fragment upstream of CC3255 (for the construction of SG19)
3256forEcoRI	ATGAATTCGCTGGCGTCCGGCCAGACC	For PCR fragment downstream of CC3255 (for the construction of SG19)
3256revNheI	ATGATGCTAGCGGTGGCGGCGTGAGCGGCC	For PCR fragment downstream of CC3255 (for the construction of SG19)
2905forEcoRI	ATGAATTCCTGCTGGCCGAGCTGGATGTC	For PCR fragment downstream of CC2906 (for the construction of SG20)
2905revNheI	ATGATGCTAGCCAAGGATGCTCTGAGCGAC	For PCR fragment downstream of CC2906 (for the construction of SG20)
2907forHindIII	AATGGAGCTTACCGACTTCGTCGACGGCTG	For PCR fragment upstream of CC2906 (for the construction of SG20)
2907revEcoRI	ATGAATTCCTCGCTGTCGAGGAACGCGCG	For PCR fragment upstream of CC2906 (for the construction of SG20)
3251forHindIII	AATGAAGCTTATGGATGTGAAAAAGGGAGG	For substitution PCR of cysteines 131 and 181 for serine
3255revNheI	ATGATGCTAGCAAGTCGACCTCGTCCAGC	For substitution PCR of cysteines 131 and 181 for serines
Cys131-P1	TCGAGCAGCCCGCGCATCT	For substitution PCR of cysteine 131 serine
Cys131-P2	AGATGCGCGGGCTGCTCGA	For substitution PCR of cysteine

		131 serine
Cys181-P1	CATAGCCCCGAGCACACCTT	For substitution PCR of cysteine 181 serine
Cys181-P2	AAGGTGTGCTCGGGGCTATG	For substitution PCR of cysteine 181 serine
Rsfxyl_r	TCAAAGCTTGCGAGACTCAAGAATTTG	Constitutive expression of CC3252 *in C. crescentus* in pJS14
SigF_3	AATTCTCGAGCTGACGGGGTTGTCACTG	Constitutive expression of CC3252 in *C. crescentus* in pJS14
lacZ3255for430	CATGAATTCCCCAGGAACGAACTCT	CC3254-*lacZ* fusion
lacZ3255for550	CATGAATTCCGTCACGCCAGCCCCTAGTTC	CC3254-*lacZ* fusion
CC3255_lacrev	AATGGTACCCCCTCCGGCGGACATG	CC3254-*lacZ* fusion
lacZ3253for360	CATGAATTCCCCGCGCACGAGGTGCCGGCG	*sigF-lacZ* fusion
lacZ3253rev1	AATGGTACCCATCAGCGCCTTCAATCGGG	*sigF-lacZ* fusion
lacZ3253rev2	AATGGTACCCATCGTGTCTTCATCGCCCG	*sigF-lacZ* fusion
SP1 CC2906	CATGCAAGGCCACAGGATAAC	5´RACE CC2907-CC2905
SP2 CC2906	CACGGCGTCGATAACGTGTAG	5´RACE CC2907-CC2905
SP3 CC2906	GACCATGAAGTTCTCGGAAATG	5´RACE CC2907-CC2905
SP1 CC3254	GAAAGAGCGATAGCGGCGGC	5´RACE CC3254
SP2 CC3254	CAGGGCGGCGGTGGTGGCGG	5´RACE CC3254
SP3 CC3254	GCGGGTCATGAGAGACTCTC	5´RACE CC3254
SP1 CC3255	GCTCGGGATCCGGGTCGGTG	5´RACE CC3254-CC3257
SP2 CC3255	GACAGCCCCACGCCATGTAG	5´RACE CC3254-CC3257
SP3 CC3255	GAGACGGGACGCCGCGCTCG	5´RACE CC3254-CC3257
RTCC0088f	GTGGCGAGAATCATGCGC	qRT-PCR CC0088
RTCC0088r	TTCATTGTGGCTGCCCG	qRT-PCR CC0088
RTCC2748f	TCAAGGGCATCAAGTCGATTG	qRT-PCR CC2748
RTCC2748r	CGTCACGGGCTGCTTCTC	qRT-PCR CC2748

RTCC2905f	CGCGCCCAACTGATCAC	qRT-PCR CC2905
RTCC2905r	GCCAGCTCCAGGTCTTTTCA	qRT-PCR CC2905
RTCC2906f	GGTTTCAACTCTCACGACCTGTT	qRT-PCR CC2906
RTCC2906r	CACCGCCATGGCCTCTT	qRT-PCR CC2906
RTCC3252f	TTATCGGCGCGGTGGTT	qRT-PCR CC3252
RTCC3252r	TGCACATCAGGACTGGTGATC	qRT-PCR CC3252
RTCC3253f	TGGAGGATCTGGTGCAAGAGA	qRT-PCR CC3253
RTCC3253r	CCCAGGTCGATCGCTTGA	qRT-PCR CC3253
RTCC3255f	TGCTCGTGGACGTCAACAAC	qRT-PCR CC3255
RTCC3255r	GGGCGCATAGCCGAGAT	qRT-PCR CC3255
RTCC3257f	CCAATCCGGCAGAACCAA	qRT-PCR CC3257
RTCC3257r	TACGTCCCGTCCGTGATGT	qRT-PCR CC3257

Table S4: Statistical analysis of the data shown in the figures.

Figure 1.

	CC3255			CC3252		
	relative expression[a]	ttest[b]	ttest[c]	relative expression[a]	ttest[b]	ttest[c]
WT no stress	1.00			1.00		
WT $K_2Cr_2O_7$	23.57	***0.003***		3.25	***0.003***	
WT $CdCl_2$	10.27	***0.001***		2.27	***0.039***	
WT H_2O_2	1.99	***0.010***		1.93	***0.000***	
WT tBOOH	1.75	***0.002***		1.46	***0.025***	
WT paraquat	1.06	0.410		1.35	***0.002***	
WT diamide	1.44	0.083		1.64	***0.001***	
Δ*sigF* no stress	0.50		***0.005***	1.48		***0.030***
Δ*sigF* $K_2Cr_2O_7$	0.43	0.211	***0.003***	1.18	0.531	***0.037***
Δ*sigF* $CdCl_2$	0.72	0.123	***0.001***	1.56	0.523	0.120
Δ*sigF* H_2O_2	0.85	***0.035***	***0.010***	2.63	***0.011***	***0.015***
Δ*sigF* tBOOH	0.35	***0.049***	***0.001***	1.18	0.144	0.149
Δ*sigF* paraquat	0.32	0.051	***0.007***	1.81	0.061	***0.003***
Δ*sigF* diamide	0.27	***0.032***	***0.014***	1.30	0.204	***0.021***

Figure 3b.

	β-galactosidase activity			ttest[c]	
	WT	Δ*sigF*	$SigF^{++}$	Δ*sigF* x WT	$SigF^{++}$ x WT
placZ290	164.18	154.74	137.96	0.693	0.261
pCKlac54-1	324.29	90.29	7478.66	***0.010***	***0.000***
pCKlac54-2	83.05	85.9	95.01	0.900	0.464
pCKlac53-1	796.34	680.83	3525.53	0.053	***0.000***
pCKlac53-2	235.43	237.55	162.91	0.954	0.080

Figure 4.

	CC2906			CC3255			CC3253		
	relative expression[a]	ttest[b]	ttest[c]	relative expression[a]	ttest[b]	ttest[c]	relative expression[a]	ttest[b]	ttest[c]
no vector no stress	1.00			1.00			1.00		
no vector $K_2Cr_2O_7$	5.66	***0.023***		7.47	0.078		2.36	***0.014***	
empty vector no stress	1.38		0.399	1.36		**0.007**	1.76		***0.003***
empty vector $K_2Cr_2O_7$	3.35	0.052	0.098	7.03	***0.011***	0.845	2.62	0.212	0.655
CC3252[++] no stress	0.91		0.338	0.48		**0.005**	1.31		0.183
CC3252[++] $K_2Cr_2O_7$	0.97	0.787	***0.019***	0.98	0.362	**0.014**	1.41	0.686	0.125

Figure 5c.

	CC2748			CC2906			CC3255		
	relative expression[a]	ttest[b]	ttest[c]	relative expression[a]	ttest[b]	ttest[c]	relative expression[a]	ttest[b]	ttest[c]
WT no stress	1.00			1.00			1.00		
WT $K_2Cr_2O_7$	25.17	***0.036***		10.26	***0.008***		25.94	***0.032***	
C131S no stress	3.43		***0.018***	4.23		***0.008***	6.70		***0.000***
C131S $K_2Cr_2O_7$	43.67	***0.032***	***0.029***	16.84	***0.033***	0.103	48.56	***0.021***	0.089
C181S no stress	8.83		***0.017***	9.73		0.059	19.96		***0.035***
C181S $K_2Cr_2O_7$	64.06	***0.001***	***0.001***	27.58	***0.020***	***0.007***	83.73	***0.005***	***0.009***
C131-181S no stress	34.25		***0.045***	22.89		***0.024***	61.63		***0.047***
C131-181S $K_2Cr_2O_7$	38.96	0.405	0.053	19.50	0.347	0.124	39.59	0.234	0.243

	CC3252			CC3253		
	relative expression[a]	ttest[b]	ttest[c]	relative expression[a]	ttest[b]	ttest[c]
WT no stress	1.00			1.00		
WT $K_2Cr_2O_7$	2.98	***0.024***		3.62	***0.003***	
C131S no stress	1.82		***0.004***	2.51		***0.000***
C131S $K_2Cr_2O_7$	6.10	0.065	0.111	10.24	0.065	0.083
C181S no stress	3.75		***0.037***	5.30		***0.047***
C181S $K_2Cr_2O_7$	13.05	0.058	***0.049***	21.87	***0.039***	***0.031***
C131-181S no stress	10.27		0.059	17.67		***0.048***
C131-181S $K_2Cr_2O_7$	7.51	0.324	0.180	14.12	0.313	***0.032***

[a] q-RT-PCR data. Values represent the fold change in expression of genes in parental or mutant strains exposed or not to the corresponding stress condition, compared to the parental strain not exposed to stress.

[b] Students' t-test. Values were calculated by comparing relative expression measured in the same strain under different growth conditions (stress x no stress). $p<0.05$ was considered as statistical significance and values are shown in bold and italic.

[c] Students' t-test. Values were calculated by comparing relative expression or β-galactosidase activity measured in different strains (mutant x wild type) under the same condition. $p<0.05$ was considered as statistical significance and values are shown in bold and italic.
